# Prediction of bifurcations by varying critical parameters of COVID-19

**DOI:** 10.1007/s11071-020-05749-6

**Published:** 2020-06-16

**Authors:** Fahimeh Nazarimehr, Viet-Thanh Pham, Tomasz Kapitaniak

**Affiliations:** 1 Biomedical Engineering Department, Amirkabir University of Technology, Tehran, 15875-4413 Iran; 2 Faculty of Electrical and Electronic Engineering, Phenikaa Institute for Advanced Study (PIAS), Phenikaa University, Yen Nghia, Ha Dong district, Hanoi, 100000 Vietnam; 3 Phenikaa Research and Technology Institute (PRATI), A&A Green Phoenix Group, 167 Hoang Ngan, Hanoi, 100000 Vietnam; 4 Division of Dynamics, Lodz University of Technology, Stefanowskiego 1/15, 90-924 Lodz, Poland

**Keywords:** SEIR model, Bifurcation, Prediction, Coupling

## Abstract

Coronavirus disease 2019 is a recent strong challenge for the world. In this paper, an epidemiology model is investigated as a model for the development of COVID-19. The propagation of COVID-19 through various sub-groups of society is studied. Some critical parameters, such as the background of mortality without considering the disease state and the speed of moving people from infected to resistance, affect the conditions of society. In this paper, early warning indicators are used to predict the bifurcation points in the system. In the interaction of various sub-groups of society, each sub-group can have various parameters. Six cases of the sub-groups interactions are studied. By coupling these sub-groups, various dynamics of the whole society are investigated.

## Introduction

Recently, many countries are dealing with coronavirus disease 2019 (COVID-19). COVID-19 is a dangerous disease with many death cases in many countries [1, 2]. COVID-19 causes respiratory illness and many other symptoms. The disease was first detected in Wuhan, China. Soon after that, it was seen in many other countries [3]. The study of epidemic outbreak in infectious disease is an interesting topic. Modeling of various biological and social facts has been a hot topic for many years [4–7]. Various methods have been used to model infectious diseases [8]. Compartmental models are useful tools in the modeling of such diseases [9, 10]. One of those models is the SEIR model. This model consists of four individuals, susceptible, exposed, infected, and resistant. This model is popular in the studies of epidemiology [11]. Many studies have been done in the modeling of COVID-19 [12]. Recently, the SEIR model has been used in [13–15]. In [16], the SIR model is used to investigate COVID-19 behavior. The prediction of COVID-19 is done in [17].

Predicting bifurcation points of the dynamical disease are important [18, 19]. Many types of research have been done to predict the bifurcation points of biological systems [19, 20]. There are some evidences that near the bifurcation points of a dynamical system, the dynamic becomes slower. It means the system needs more time to pass the transients and reach its final state [21]. Various methods have been used in the prediction of bifurcation points. Some of the well-known indicators are autocorrelation at lag-1 and variance [22]. They bear some issues in predicting bifurcations in more complex dynamics. Recently, a new method has been introduced to improve the conventional indicators [20]. Lyapunov exponent is another interesting predictor of bifurcation points [23, 24].

The study of networks of systems can help to understand the collective behavior of systems. Complex networks have a great impact on human life [25]. They have many components which are interacting with various connections [26, 27]. Synchronization is one of the most interesting properties in the dynamics of networks [28, 29]. Many biological systems, such as neurons, do their task in a network [30, 31]. Many studies have been done on networks [32, 33]. A chaotic model of epilepsy based on the neural network was proposed in [34]. Various dynamics of a multilayer network was discussed in [35]. In this paper, various dynamics of the SEIR system, as a model for the development of COVID-19, are investigated. Bifurcation diagrams of the system are studied to show its various behaviors by changing parameters. Bifurcation points of the system are discussed, and they are predicted using autocorrelation. Also, the interaction of five cities is studied with various connections and parameters to show the effect of traveling in the outbreak of the disease.

## Studied model

**Fig. 1:**
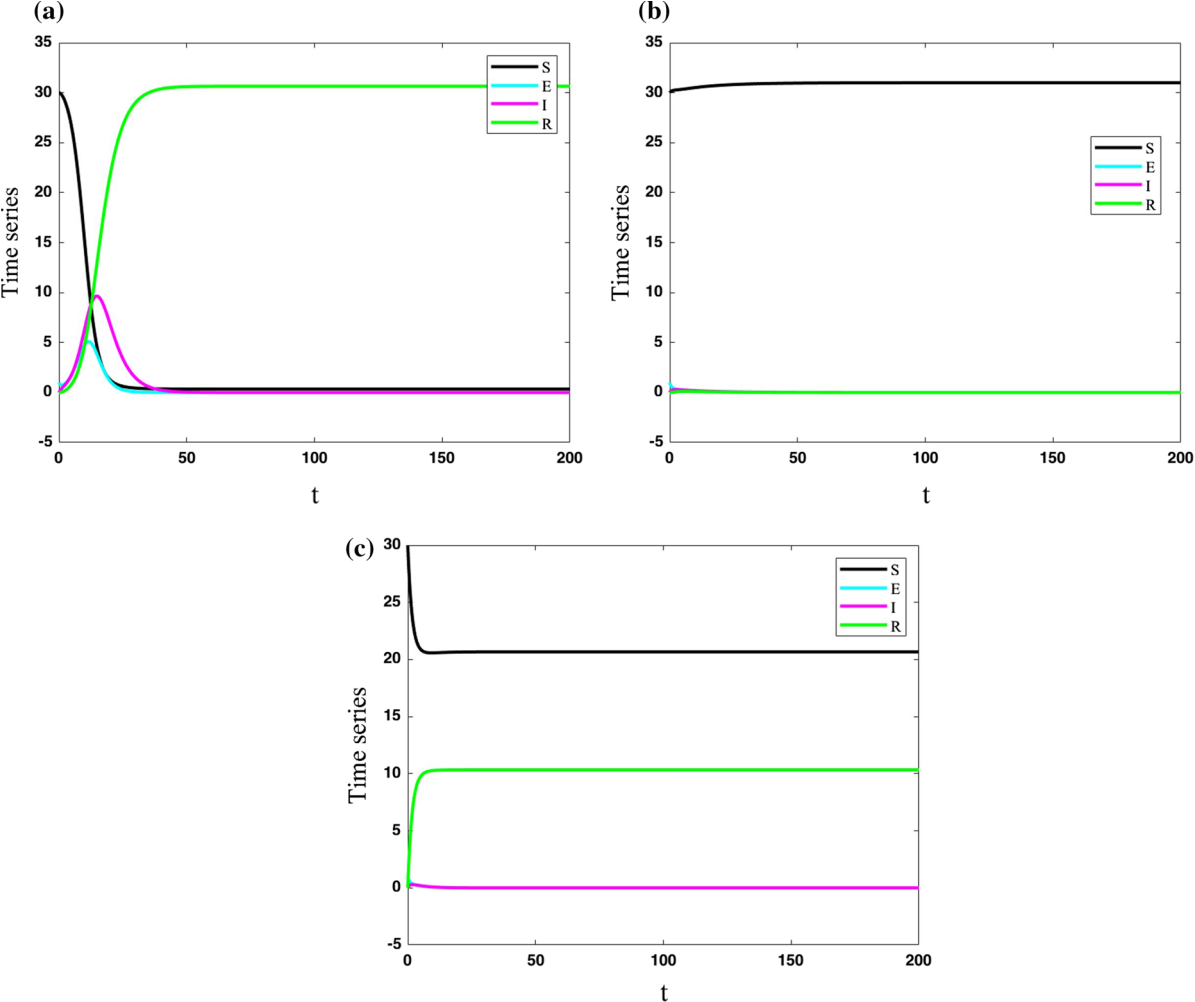
Three strategies of the outbreak with various parameters; **a** in parameters $(\mu ,\beta ,\nu ,\sigma ,\gamma ) = (0,0.9,0,0.5,0.2)$ and initial conditions $(S_{0},E_{0},I_{0},R_{0})=(30,1,0,0)$; **b** in parameters $(\mu ,\beta ,\nu ,\sigma ,\gamma ) = (0.4,0.9,0,0.5,0.2)$ and initial conditions $(S_{0},E_{0},I_{0},R_{0})=(30,1,0,0)$; **c** in parameters $(\mu ,\beta ,\nu ,\sigma ,\gamma ) = (0.4,0.9,0.2,0.5,0.2)$ and initial conditions $(S_{0},E_{0},I_{0},R_{0})=(30,1,0,0)$; By increasing the background of mortality, all the people become susceptible, and the number of other groups goes to zero. Also, the increase in the vaccination rate increases the number of resistant people

In this paper, the SEIR model is used as a model for the development of COVID-19. It is a compartmental model of infectious disease and its epidemic outbreak [11]. It contains four classes of people as susceptible (S), exposed (E), infected (I), and resistant (R). The SEIR model is as follows:

1 $$\begin{aligned} \begin{array}{l} \dot{S}=\mu (N-S)-\frac{\beta SI}{N}-\nu S\\ \dot{E}=\frac{\beta SI}{N}-(\mu +\sigma ) E\\ \dot{I}=\sigma E-(\mu +\gamma ) I\\ \dot{R}=\gamma I-\mu R+\nu S\\ \end{array} \end{aligned}$$

where $N=S+E+I+R$. In this model *S*, *E*, *I*, *R* are the number of susceptible, exposes, infected, and resistant people. Parameters $\mu ,\beta ,\nu ,\sigma ,\gamma$ are the background of mortality without considering the disease state, the speed of moving people from susceptible to exposed, the vaccination rate, the speed of moving people from exposed to infected, and the speed of moving people from infected to resistance. It should be noted that the vaccination causes people to move from a susceptible group to the resistant one directly. The model can show various dynamics of the epidemic outbreak of COVID-19 [13–15]. The system is solved for 200 days. Three strategies of the outbreak with various parameters are shown in Fig. 1. The set of parameters are $(\mu ,\beta ,\nu ,\sigma ,\gamma )=(0,0.9,0,0.5,0.2)$, $(\mu ,\beta ,\nu ,\sigma ,\gamma )=(0.4,0.9,0,0.5,0.2)$, $(\mu ,\beta ,\nu ,\sigma ,\gamma )=(0.4,0.9,0.2,0.5,0.2)$ for parts (a), (b), and (c) of the figure, respectively. Initial conditions are taken as $(S_{0},E_{0},I_{0},R_{0})=(30,1,0,0)$. It can be seen that by increasing the background of mortality, all the people become susceptible, and the number of other groups goes to zero. Also, the increase in the vaccination rate increases the number of resistant people.

## Dynamical properties

**Fig. 2:**
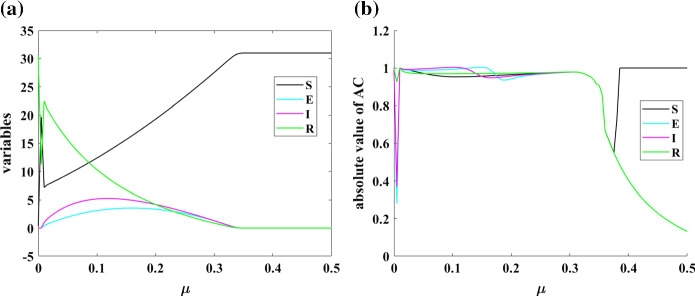
**a** Bifurcations of Model (1) by changing parameter $\mu$; **b** Absolute value of autocorrelation of dynamics of Model (1) by changing parameter $\mu$; Dynamics of the system have various bifurcation points. The AC approaches one in bifurcation points

To study the dynamical properties of the model, various bifurcations are investigated. Part (a) of Fig. 2 shows the bifurcations of Model (1) by changing the parameter of the background of mortality without considering the disease state. It should be considered that in each parameter, the variables approach to an equilibrium point by passing enough time. The bifurcation diagram is plotted with the forwarding continuation method. Dynamics of the system have various bifurcation points. To predict bifurcation points of the model, autocorrelation (AC) is used. The absolute value of autocorrelation determines the slowness of dynamics by approaching to “one” near the bifurcation points. Autocorrelation is calculated from the time series of susceptible, exposed, infected, and resistant variables, which are shown in part (b) of Fig. 2. It can be seen that the AC approaches one in bifurcation points $\mu =0.01$ and $\mu =0.34$. Also, the population of exposed and infected people has a change of concavity in approximately $\mu =0.1$, and the AC can predict this bifurcation. The AC of the resistant population is fixed to one in $\mu >0.4$ because of a computational error. There are not any transients in the time series of $\mu >0.4$ with a forward continuation method.

**Fig. 3:**
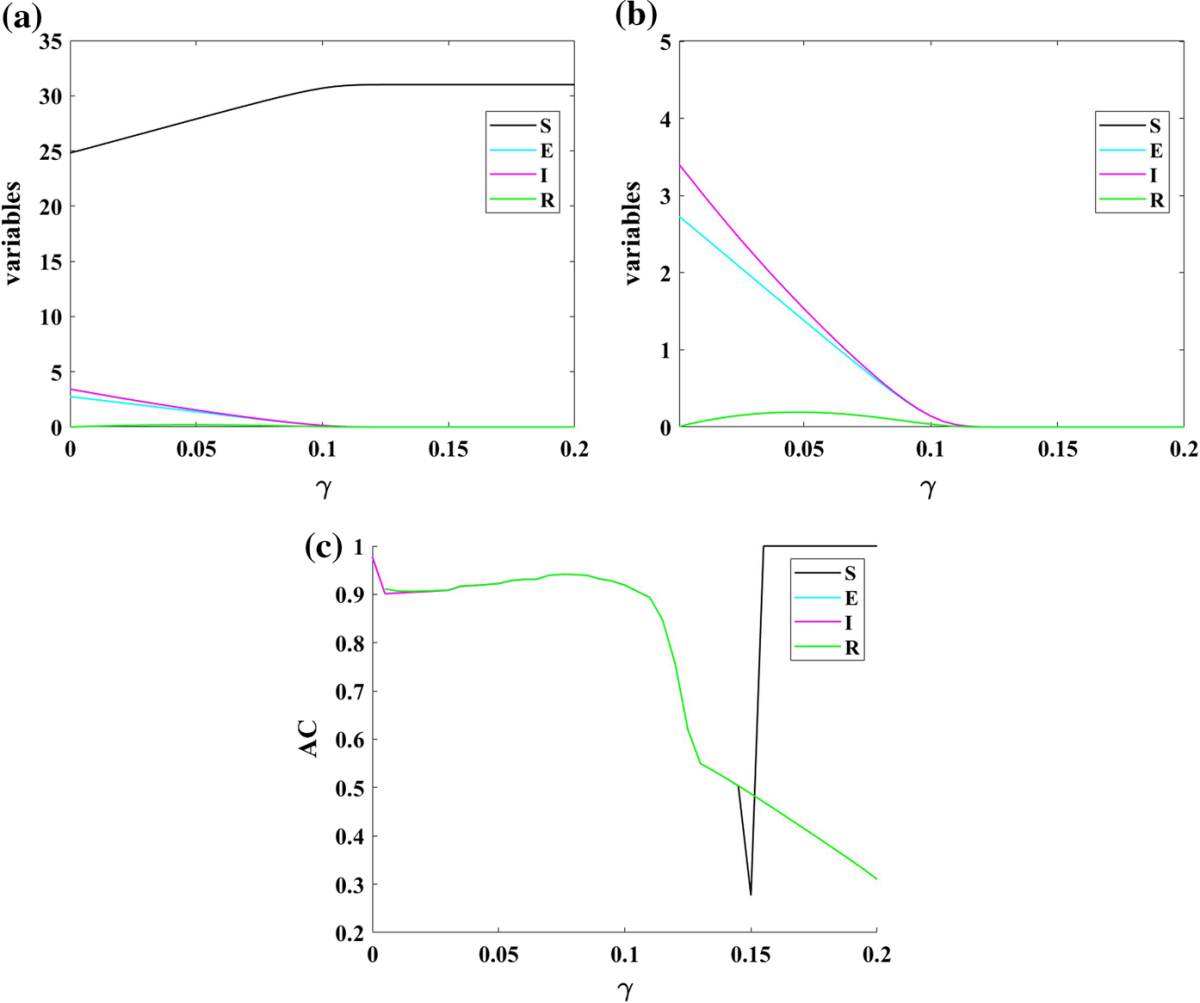
**a** Bifurcation diagram of Model (1) for changing parameter $\gamma$; **b** The zoomed view of bifurcation diagram; **c** Autocorrelation of dynamics of Model (1) by changing parameter $\gamma$; AC predicts bifurcation point in $\gamma = 0.105$ and also shows a small peak in the change of concavity of resistant population

The speed of moving people from infected to resistance is another parameter that is investigated in the following. Figure 3 shows the bifurcation diagram of Model (1) concerning changing parameter $\gamma$. In part (a) of that figure, bifurcation diagram of four variables of Model (1) by changing parameter $\gamma$ is plotted. To have a better view of the three smaller variables, their zoomed view is shown in part (b). AC is used to predict the bifurcation points of the population variables as part (c). AC predicts bifurcation point in $\gamma =0.105$ and also shows a small peak in the change of concavity of resistant population. Predicting these bifurcation points can help the world from being surprised by variations of COVID-19 epidemic states.

## Interaction of various sub-groups of society

**Fig. 4:**
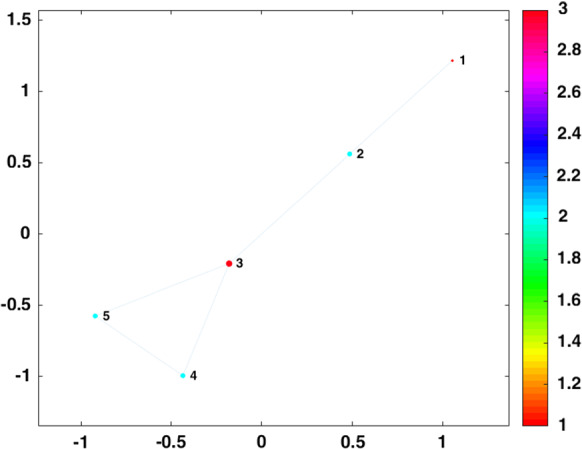
Watts–Strogatz graph with five nodes, mean node degree 2, and rewriting probability 0.15. The size and color of each node are proportional to its degree. The node degree 3 is shown in red, and the node degree 1 is shown in orange color. The node degree related to each color is shown in the color bar. (Color figure online)

In this section, the connections of five cities are considered to show the effect of intercity traffic restrictions. To reach this goal, System (1) is considered as the model of development of COVID-19 in each city. Then, the five cities are connected in a Watts–Strogatz network [36, 37]. The connection graph is shown in Fig. 4. So, each of the four variables of System (1) is connected to the variables of other cities as Eq. 2. $x_{i}$ is the state variables of each city, and *f* is their functions. The nodes are connected by coupling matrix *C*, and $\lambda$ is the strength of coupling. An undirected network is used. So, we consider if the coupling between node A and node B is one, it means that people can travel from city A to B and vice versa.

2 $$\begin{aligned} \dot{x_{i}}=f(x_{i})+\lambda \sum _{j=1}^{5} c_{ij}x_{j} \, i=1,2,\ldots ,5 \end{aligned}$$

Now, various cases are studied. The initial values of variables are set constant as Table 1.

**Table 1:** Initial values of variables for the five cities

Nodes	$S_0$	$E_0$	$I_0$	$R_0$
1	9	13	0	31
2	5	3	12	6
3	15	11	30	29
4	2	23	8	13
5	17	29	13	30

**Fig. 5:**
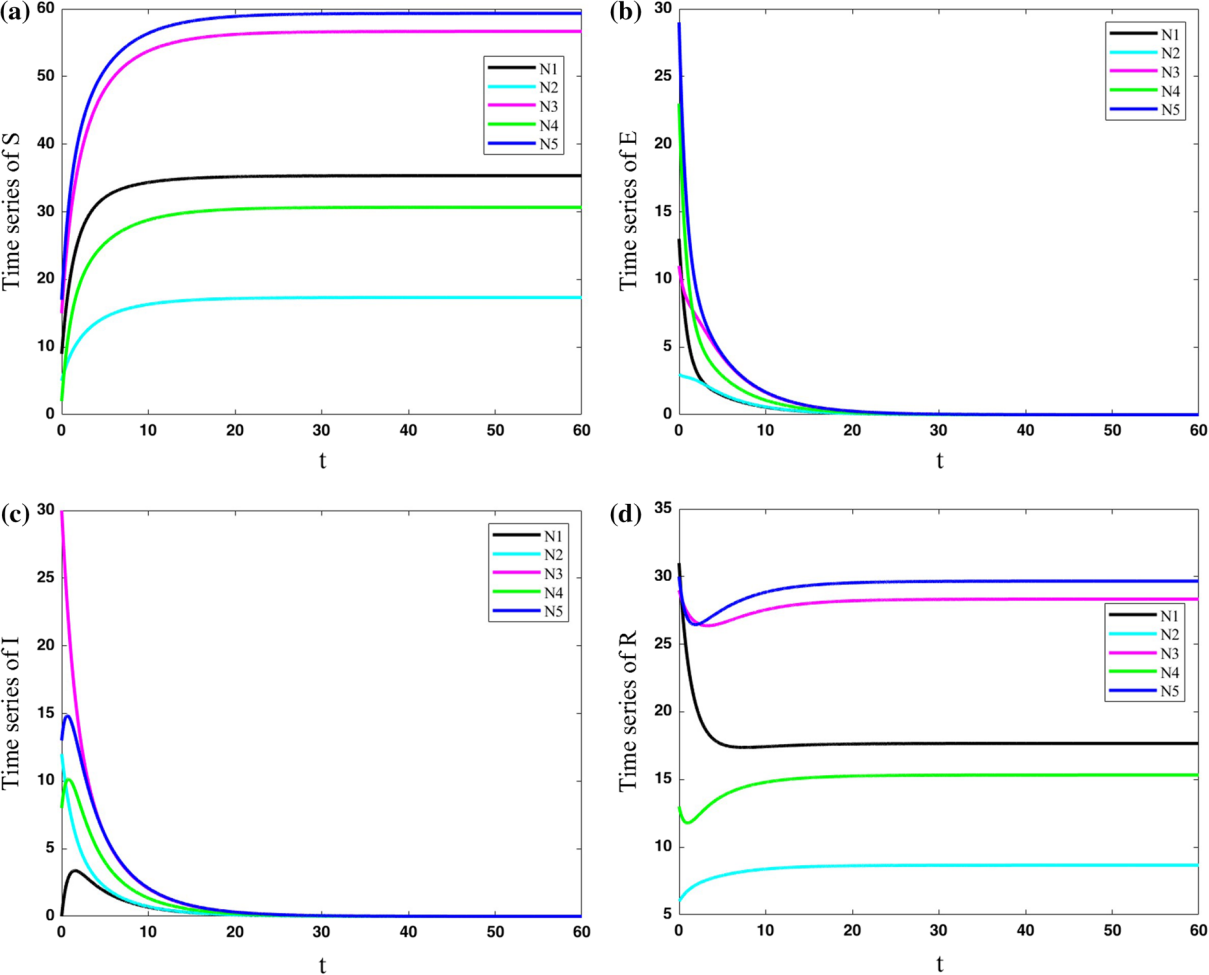
Variations of state variables of five cities with the same parameters for cities as $(\mu ,\beta ,\nu ,\sigma ,\gamma ) = (0.4,0.9,0.2,0.5,0.2)$ and coupling strength $\lambda =0$; Each city has its own evolution, and they have no effect on each other. The number of exposed and infected people approaches zero by passing the time, and the number of susceptible and resistant people becomes constant but different for each city

In the first case, we consider that no people can travel from one city to another one ($\lambda =0$). Parameters are set constant for all five cities as $(\mu ,\beta ,\nu ,\sigma ,\gamma ) = (0.4,0.9,0.2,0.5,0.2)$. The results of the population variables of the five nodes are shown in Fig. 5. The results show that each city has its own evolution, and they have no effect on each other. The number of exposed and infected people approaches zero by passing the time, and the number of susceptible and resistant people becomes constant but different for each city.

**Fig. 6:**
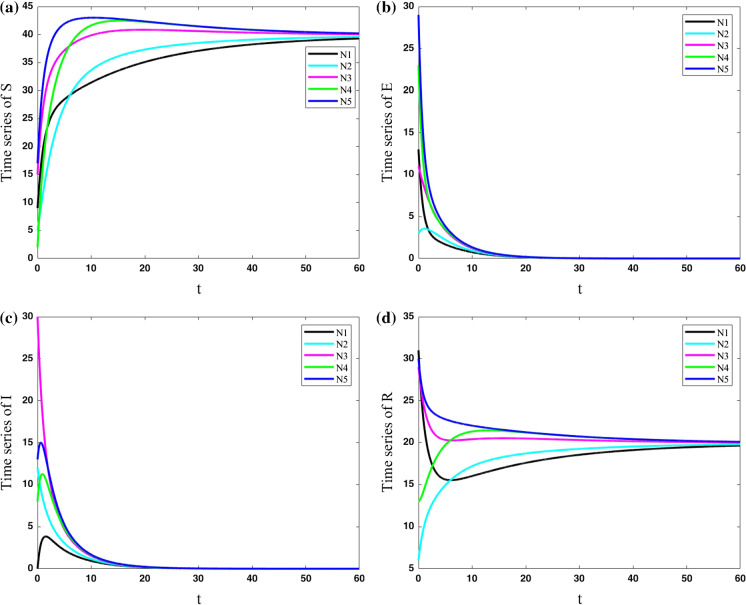
Variations of state variables of five cities with the same parameters for cities as $(\mu ,\beta ,\nu ,\sigma ,\gamma ) = (0.4,0.9,0.2,0.5,0.2)$ and coupling strength $\lambda =0.1$; This small connection makes all the cities the same in the evolution of COVID-19. However, their approach to the same value is slow

In the second case, the cities are considered to have some connections to each other ($\lambda =0.1$). Parameters are set constant for all five cities as $(\mu ,\beta ,\nu ,\sigma ,\gamma ) = (0.4,0.9,0.2,0.5,0.2)$. The results which are shown in Fig. 6 present that this small connection makes all the cities become the same in the evolution of COVID-19. However, their approaches to the same value are slow.

**Fig. 7:**
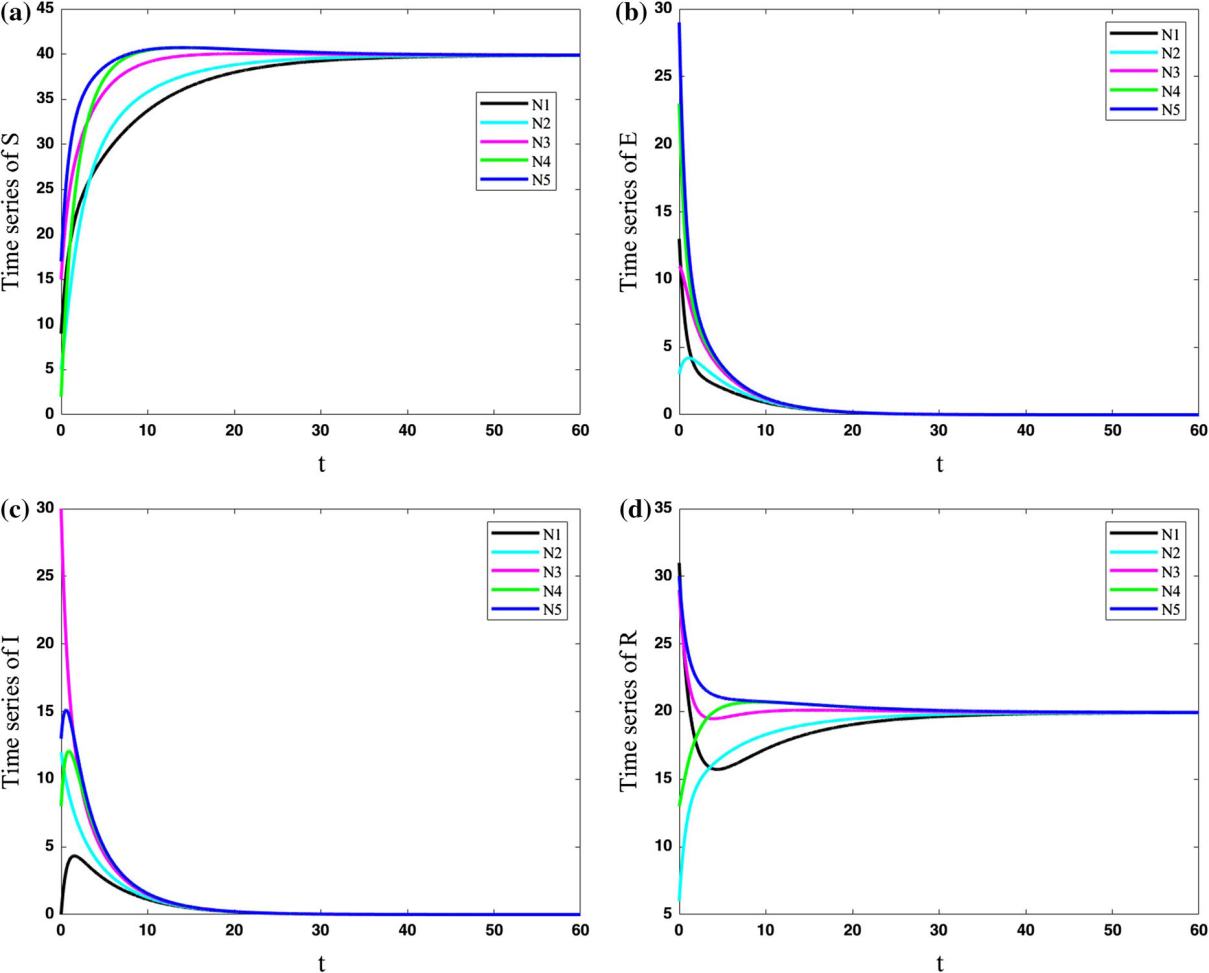
Variations of state variables of five cities with the same parameters for cities as $(\mu ,\beta ,\nu ,\sigma ,\gamma ) = (0.4,0.9,0.2,0.5,0.2)$ and coupling strength $\lambda =0.2$; In this network, by passing enough time, the five cities become synchronized

In the third case, the cities are considered to have more significant connections to each other ($\lambda =0.2$). Parameters are set constant for all five cities as $(\mu ,\beta ,\nu ,$ $\sigma ,\gamma ) = (0.4,0.9,0.2,0.5,0.2)$. The results of this network are shown in Fig. 7. In a comparison of this network with the previous one, the current network is faster in approaching their same constant values. In other words, by passing enough time, the five cities become synchronized.

**Fig. 8:**
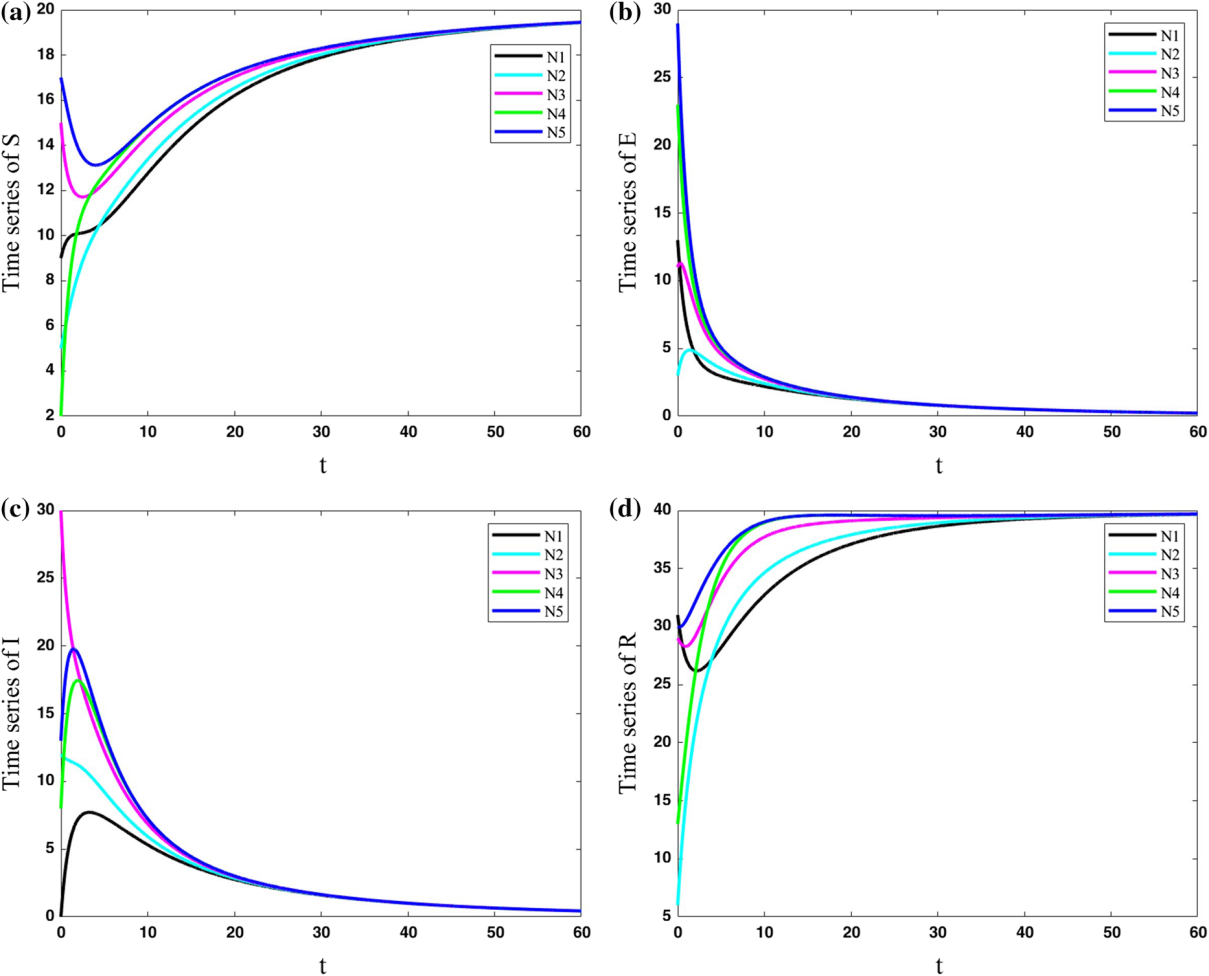
Variations of state variables of five cities with the same parameters for cities as $(\mu ,\beta ,\nu ,\sigma ,\gamma ) = (0.1,0.9,0.2,0.5,0.2)$ and coupling strength $\lambda =0.2$; Decreasing the parameter $\mu$ makes an undershoot in susceptible and resistant population. Also, the lower $\mu$ parameter makes the susceptible population constant to a lower number and resistant population to a higher number

In the fourth case, the cities are considered to have some connections to each other ($\lambda =0.2$). In the study of a single system, the results show that parameter $\mu$ can change the dynamic of the evolution of COVID-19. Parameters of the network are set constant for all five cities as $(\mu ,\beta ,\nu$ $,\sigma ,\gamma ) = (0.1,0.9,0.2,0.5,0.2)$ in this case. The parameter $\mu$ is different from the previous network. The results (which are presented in Fig. 8) show that decreasing the parameter $\mu$ makes an undershoot in susceptible and resistant population. Also, the lower $\mu$ parameter makes the susceptible population constant to a lower number and resistant population to a higher number.

To have a more realistic viewpoint, the parameters of cities are considered to be different. In the next two cases, parameters for each city are considered as, $N_{1}: (\mu ,\beta ,\nu ,\sigma ,\gamma ) = (0.4,0.9,0.2,0.5,0.2)$, $N_{2}: (\mu ,\beta ,\nu ,\sigma ,\gamma ) = (0.1,0.5,0,0.1,0.01)$, $N_{3}: (\mu ,\beta ,\nu ,\sigma ,\gamma ) = (0,0.2,0.05,$ 0.05, 0.1), $N_{4}: (\mu ,\beta ,\nu ,\sigma ,\gamma ) = (0.2,0.7,0.13,0.45,0.15)$, $N_{5}: (\mu ,\beta$ $,\nu ,\sigma ,\gamma ) = (0.33,0.66,0.11,0.2,0.07)$.

**Fig. 9:**
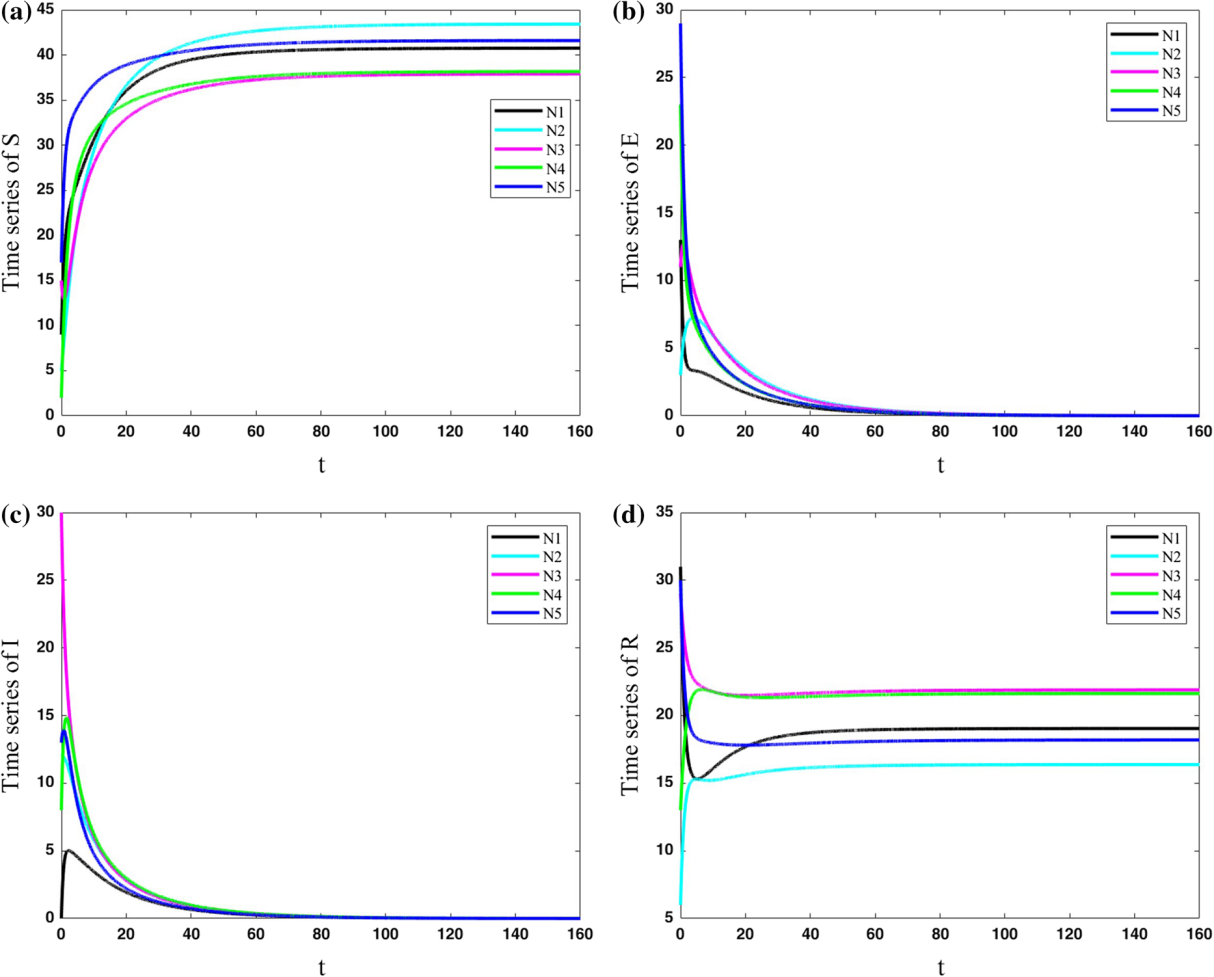
Variations of state variables of five cities with different parameters for cities and coupling strength $\lambda =0.2$; The population of the susceptible and resistant group in the cities cannot be synchronized, and they are different in all the times. However, the population of exposed and infected people approaches to zero in all the cities

In the fifth case, the cities with various parameters are considered to have some connections to each other ($\lambda =0.2$). The results of Fig. 9 show that in the case with various parameters for each city, the population of the susceptible and resistant group in the cities cannot be synchronized, and they are different in all the times. However, the population of exposed and infected people approaches to zero in all the cities.

**Fig. 10:**
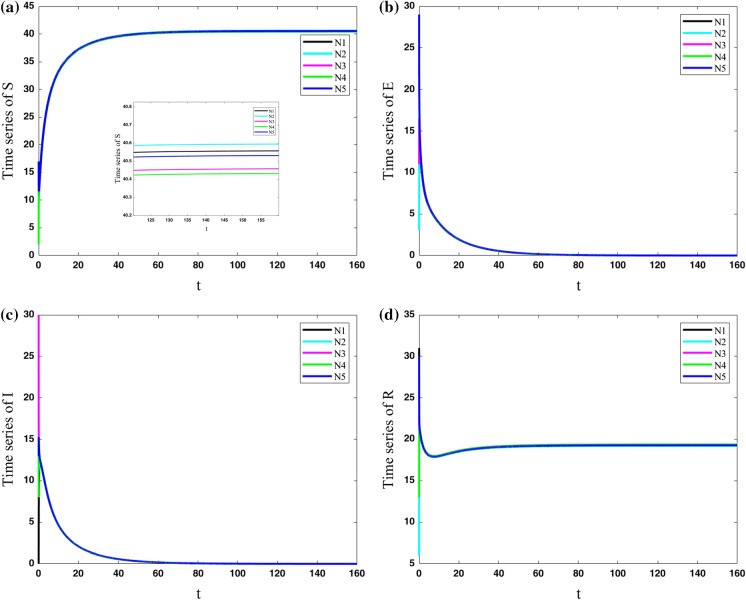
Variations of state variables of five cities with different parameters for cities and coupling strength $\lambda =11$; The population of susceptible and resistant people of various cities is approximately synchronized; however, the zoomed view of the susceptible population shows that they are not completely synchronized

In the sixth case, the cities with various parameters are considered to have more connections to each other ($\lambda =11$). In other words, the coupling strength is increased to reveal the parameter in which the dynamics of various cities become synchronized. The results of the network are shown in Fig. 10. It shows that the population of exposed and infected people in all cities approaches zero by passing the time. The population of susceptible and resistant people of various cities is approximately synchronized; however, the zoomed view of the susceptible population shows they are not completely synchronized.

So, in cities with the same parameters, increasing the coupling strength causes the dynamics of five cities to become the same by passing enough time. However, in cities with various parameters, reaching the same dynamics needs a much bigger coupling strength. Also, decreasing parameter $\mu$ changes the dynamics of COVID-19 outbreak.

## Conclusion

In this paper, the SEIR model was used as the model for the development of COVID-19 outbreak. In the first step of this study, various bifurcations of the model by changing some critical parameters such as the background of mortality without considering the disease state and the speed of moving people from infected to resistance were discussed. Then, autocorrelation was used to predict bifurcation points of the model. To visualize the collective behavior of COVID-19 by the effect of traveling between various cities, a network consisting of five cities was studied. The effect of various parameters and the coupling strength between cities was discussed. Watts–Strogatz network was used to simulate the connection of five cities. In the interaction of various cities, each city can have various parameters. By coupling these cities, various dynamics of the whole society were investigated. Six cases were defined to study various cases of cities interactions. The results showed that in cities with the same parameters, by increasing the coupling strength from zero, the dynamics of five cities become the same by passing enough time. However, in cities with various parameters, the same dynamics cannot be reached very quickly, and it needs a much bigger coupling strength. Also, the results showed that decreasing the parameter $\mu$ can change the dynamics of COVID-19 outbreak in cases with the same parameters for five cities.
